# Adolescent birth rates and the urban social environment in 363 Latin American cities

**DOI:** 10.1136/bmjgh-2022-009737

**Published:** 2022-10-17

**Authors:** Ariela Braverman-Bronstein, Dèsirée Vidaña-Pérez, Ana F Ortigoza, Laura Baldovino-Chiquillo, Francisco Diez-Canseco, Julie Maslowsky, Brisa N. Sánchez, Tonatiuh Barrientos-Gutiérrez, Ana V. Diez Roux

**Affiliations:** 1 Urban Health Collaborative, Dornsife School of Public Health, Drexel University, Philadelphia, Pennsylvania, USA; 2 Center for Survey Research and Evaluation, National Institute of Public Health, Cuernavaca, Mexico; 3 School of Medicine, Universidad de los Andes, Bogota, Colombia; 4 CRONICAS Center of Excellence in Chronic Diseases, Universidad Peruana Cayetano Heredia, Lima, Peru; 5 Center of Excellence in Maternal and Child Health School of Public Health, University of Illinois at Chicago, Chicago, Illinois, USA; 6 Center for Population Health Research, National Institute of Public Health, Cuernavaca, Mexico

**Keywords:** public health, maternal health, epidemiology

## Abstract

**Introduction:**

Latin America has the second-highest adolescent birth rate (ABR) worldwide. Variation between urban and rural areas and evidence linking country development to ABR points towards upstream factors in the causal pathway. We investigated variation in ABR within and between cities, and whether different features of urban social environments are associated with ABR.

**Methods:**

We included 363 cities in 9 Latin American countries. We collected data on social environment at country, city and subcity levels and birth rates among adolescents (ages 15–19). We investigated variation in ABR within and between countries and cities along with associations between social environment and ABR by fitting three-level negative binomial models (subcities nested within cities nested within countries).

**Results:**

The median subcity ABR was 58.5 per 1000 women 15–19 (IQR 43.0–75.3). We found significant variability in subcity ABR between countries and cities (37% of variance between countries and 47% between cities within countries). Higher homicide rates and greater population growth in cities were associated with higher ABR (rate ratio (RR) 1.09; 95% CI 1.06 to 1.12 and RR 1.02; 95% CI 1.00 to 1.04, per SD, respectively), while better living conditions and educational attainment in subcities were associated with lower ABR after accounting for other social environment characteristics (RR 0.95; 95% CI 0.92 to 0.98 and 0.78; 95% CI 0.76 to 0.79, per SD, respectively).

**Conclusions:**

The large heterogeneity of ABR found within countries and cities highlights the key role urban areas have in developing local policies. Holistic interventions targeting education inequalities and living conditions are likely important to reducing ABR in cities.

**WHAT IS ALREADY KNOWN ON THIS TOPIC**

- Latin America, one of the most urbanised regions of the world, is the region with the second-highest adolescent birth rate (ABR). Abundant research on individual-level determinants of adolescent pregnancy consistently points towards the importance of education and socioeconomic conditions and development. Evidence on neighbourhood features associated with ABRs in high-income countries also suggests that neighbourhood socioeconomic conditions are important determinants, but this research is scarce in low-income and middle-income countries.

**WHAT THIS STUDY ADDS**

- Our study is among the first to investigate city and subcity differences in ABR and the factors associated with this variability. Our results highlight the large variation of ABRs between and within countries in cities in Latin America. Furthermore, they suggest that features of the urban social environment have important associations with ABR. Specifically higher city levels of violence were associated with higher ABR whereas better living conditions and educational attainment were associated with lower ABR.

**HOW THIS STUDY MIGHT AFFECT RESEARCH, PRACTICE OR POLICY**

- The large heterogeneity found within countries and cities highlights the need to prioritise the development of local policies to reduce inequalities in cities. Our findings also support the need to develop context-based interventions considering the city and subcity social environments to reduce ABRs and reach the Sustainable Development Goals.

## Introduction

Although in many cases births to adolescents can reflect the legitimate desires or aspirations of young women, it is also true that births to adolescents (especially young adolescents) are associated with poor birth outcomes and can limit the educational and professional opportunities of women.^1^ Adolescent birth rate (ABR) is a progress indicator for the Sustainable Development Goals (SDG) under the healthcare target.^2^ In 2020, the ABR worldwide was 41.2 per thousand women 15–19 years old,^3^ with 95% of these births occurring in low-income and middle-income countries (LMICs).^1^ Latin America (LA) and the Caribbean are the region with the second-highest ABR in the world (60.7 births per 1000 women 15–19 years), second only to sub-Saharan Africa (100.5 births per 1000 women 15–19 years).^3^ Despite the implementation of interventions/policies targeted at reducing ABR, including national adolescent pregnancy prevention programmes, LA has experienced a less favourable evolution of ABR compared with other regions.^4^ Moreover, evidence shows major differences in ABR across countries and social groups in the region, pointing to the role of upstream factors in the mechanisms leading to adolescent pregnancy.^5^

To date, most descriptions of ABR in the region have used data at a national or subnational level with less focus on the urban social environment. Furthermore, most research has focused on individual-level factors related to reproductive health and education, with less focus on the upstream factors that may influence cultural and social decisions related to adolescent behaviour in LA.^6 7^ A cross-country study of ABR from 1990 to 2012 (including LA and other regions), found that rates declined in countries that experienced greater economic growth and reductions in income inequality, which may be explained by the increase in employment and education opportunities.^8^ Other evidence from national surveys suggests that lower individual education levels and socioeconomic status are associated with higher ABR,^9^ suggesting that social and economic conditions are important upstream determinants of ABR. More broadly, socioeconomic and political contexts have been identified as social determinants of adolescent health.^10^

LA is one of the most urbanised regions of the world.^11^ This urbanisation is accompanied by large income and social inequalities.^12^ Urban social environments (including living conditions, employment opportunities, educational attainment and contextual violence) are heterogeneous across cities within the same country, and even neighbourhoods within cities can have very different contextual and economic features.^13^ While there is significant evidence on the importance of social determinants for adolescent health outcomes,^14^ there is little research on how features of urban environments relate to ABR in LMIC. Evidence from high-income countries (HICs) suggests that neighbourhoods with worse socioeconomic conditions have higher ABR compared with neighbourhoods with better socioeconomic conditions.^15 16^ Other urban social factors, such as neighbourhood crime and violence, significantly impact health and adolescent well-being.^17^ Fear of being a victim and consistent exposure to violence have been linked to mental health problems such as anxiety and depression, affecting overall adolescent well-being.^18^ The large heterogeneity in urban environments across and within LA cities provides a unique opportunity to identify what features of urban social environments are associated with ABR in the growing cities of LMIC. This characterisation is important to identify targets for policies and interventions to reduce ABR which could amplify the benefits of interventions focused on individual behavioural change.

Based on a social determinant of health framework,^19^ we identified selected features of the urban social environment that have been found to be associated with adolescent sexual and reproductive health outcomes at a national or at a more disaggregated level (online supplemental figure 1). Using a harmonised dataset including country, city and subcity characteristics from 363 cities in 9 LA countries, we investigated variation in ABR within and between cities. In addition, we examined whether features of urban social environments defined at the city or subcity level are associated with ABR.

10.1136/bmjgh-2022-009737.supp1 Supplementary data

## Methods

Data were drawn from the Salud Urbana en America Latina Project (SALURBAL), which has compiled and harmonised health, social and built-environment data from 371 cities (population ≥1 00 000 in 2010) in 11 countries.^20^ Each city is composed by administrative subunits (ie, municipios, comunas, distritos, partidos, delegaciones, cantones or corregimientos) which we will refer to as subcities. This study includes all subcity units that had available information from vital statistics registries from 2014 to 2016. We included 1403 subcities from 363 cities in 9 countries: Argentina, Brazil, Chile, Colombia, Costa Rica, Guatemala, Mexico, Panama and Peru.

The outcome of interest was ABR, defined as the total number of births per 1000 women aged 15–19 years. We assessed ABR at the subcity level to capture heterogeneity within larger cities composed of multiple subcity units (n=183 cities). We pooled data for years 2014–2016 retrieved from vital statistics registration from each country and linked to city/subcity levels based on the mother’s residential location at the time of birth. The population of female adolescents 15–19 years living in each subcity was retrieved using census-derived population projections for each country. After estimating the rates, this variable was categorised in quartiles for the descriptive analysis, with the first quartile corresponding to the lowest rates.

We examined a selected set of predictors at the country, city and subcity level. The level of measurement was determined by the substantive meaning of the predictor itself (ie, the construct of interest), the level at which variability in the construct was present -some indicators had important variability at a subcity level while others did not vary that much- and the data availability. The key country-level measure was ‘unmet contraceptive needs’ (UCN) assessed by the per cent of UCN for women of reproductive age (15–45 years). This indicator, used to monitor family planning programmes, refers to the proportion of married women 15–44 who do not want to become pregnant but are not using contraception.^21^ Country-specific percentages were retrieved from the World Bank Database.^22^ This information is collected from nationally representative demographic or health surveys, so geographically disaggregated data for specific cities was unavailable.

City-level variables included average population size, population growth, gross domestic product per capita (GDP), homicide rates and coverage of the first triple viral (MMR1, measles-mumps-rubella) vaccine dose. Population size was the average population in 2014–2016 using population counts for each city. Population growth was defined as the city-specific per cent change in population from 2010 to 2015. These variables were estimated using country-specific population projections generated by each country’s Census Bureau. GDP for each city for year 2015 was derived from modelled estimates at the subnational level.^23^ For Costa Rica, the data represents GDP at the national level. We estimated homicide rates for the 2014–2016 period as a proxy for city violence using previously harmonised mortality data by SALURBAL. We calculated the count of deaths with the International Classification of Diseases (ICD) codes corresponding to intentional injury due to violence (ICD10 codes: X85-Y09, Y871) by city and divided the count by the total city population and multiplied by 100 000. As a proxy for city healthcare access, we included the coverage of MMR1 vaccine first dose, defined as the percentage of administered doses from the total estimated number of doses to be applied among the population of 1 year old in 2016 obtained from the WHO.^24^

Subcity-level exposures included three scores characterising the social and economic environment. These scores (living conditions, service provision and educational attainment) were developed by SALURBAL using principal component analysis and were found to be related to city infant mortality in prior work.^25^ The living conditions score includes: (1) per cent of households with piped water inside the dwelling; (2) per cent of households with overcrowding (more than three people per room) and (3) per cent of population aged 15–17 years attending school (range −10.6 to 4.03). The service provision score includes: (1) per cent of households with access to water from a municipal public/private water network and (2) per cent of households with sewage system connected to a municipal public/private sewage network (range −4.61 to 2.17). The educational attainment score includes: (1) per cent of population aged 25 years or above that has completed high school level or above and (2) per cent of population aged 25 years or above that completed university level or above (range −3.43 to 7.37). For the three measures, higher values indicate better socioeconomic environment.

### Statistical analysis

We present the distribution of ABR by city and country as well as the distribution of the exposure variables by quartiles of subcity ABR. To examine the variability across subcities, within cities and across cities within the same country we fitted a three-level negative binomial model with random intercepts for cities and countries (subcities nested within cities nested within countries). To assess the degree of clustering within cities and countries we estimated intraclass correlations coefficients (ICC) based on the formula for negative binomial models described by Oliveira *et al*.^26^ Given that the mean and variance for the negative binomial distribution are related, the ICC was calculated holding the mean ABR constant as the overall mean ABR across subcities.

To assess the associations of country, city and subcity-level exposures with ABR, we fitted a series of sequential three-level negative binomial models: model 1 included each city or subcity level variable separately along with a random intercept for each city and country; model 2 included UCN (only the country-level variable); model 3 added all the city-level variables to model 2 and model 4 added all the subcity-level variables to model 3. This modelling sequence allowed us to examine how coefficients and the random effects variances changed as variables from different levels were added.

We conducted a sensitivity analysis in a subset of 176 subcities that had data available for the % of women 26–49 that had a pap smear test in the last 3 years as an alternate and more disaggregated indicator of reproductive healthcare access. This variable was harmonised by the SALURBAL project and city-level prevalence estimates were derived using Empirical Bayes smoothing techniques.^27 28^

When examining crude associations, we did not find evidence of non-linear associations by visual exploration, thus, exposure variables were treated as continuous. We also tested for collinearity between predictors, finding that the correlations between exposures ranged from a high of 0.37 for population size average and GDP to a low of 0.02 for population growth and service provision score. All predictors were standardised to a mean of 0 and an SD of 1 before fitting the models. The outcome was operationalised as number of births per 1000 women 15–19 years. We established a significance level of 0.05 and all analyses were done using Stata V.16 and R V.3.9.1.

## Results

**Figure 1:**
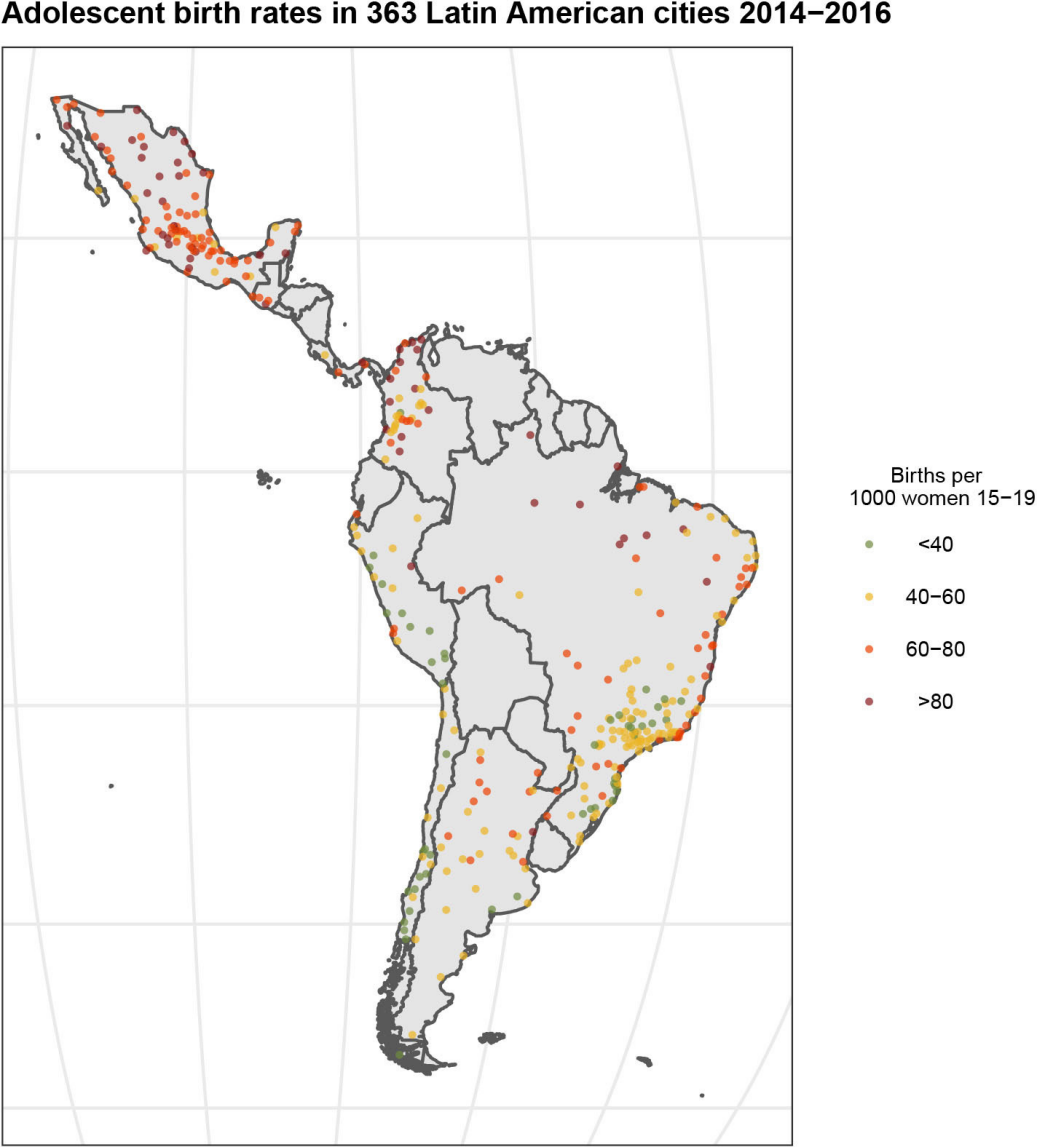
Distribution of adolescent birth rates in 363 Latin American cities (2014–2016). Each dot represents each one of the 363 cities included in the study. City-level adolescent birth rates were categorised in quartiles where read represents the highest quartile and green the lowest.

The median ABR across all 1403 subcities included in the analyses was 58.5 births per 1000 women 15–19 yearsd (IQR 43.0–75.3), The median ABR for each country was: Argentina 54.3; Brazil 53.4; Chile 33.8; Colombia 57.9; Costa Rica 37.8; Guatemala 69.8; Mexico 69.4; Panama 83.6 and Peru 35.3 births per 1000 women 15–19. Figure 1 shows the distribution of the city-level ABRs, the median city ABR was 55.2 births per 1000 women 15–19 (IQR 45.1–68.6) across the 363 cities. Although there was important heterogeneity across countries, there was also substantial variability within countries. We observe some clustering where lower city-ABR concentrate in countries with lower median ABR (Peru, Chile and Brazil) while higher city-ABR concentrate in Mexico, Guatemala and Panama, with the highest city ABR median being in Panama (86.3) and the lowest in Chile (33.8) (online supplemental figure 2). The ICC estimation showed that 37% of the variability occurs between countries and 47% between cities within countries. Country, city and subcity-level characteristics by subcity ABR quartile are shown in table 1. In general, subcities in countries with a higher percentage of UCN were found within the two highest ABR quartiles, while subcities in countries with lower percentage of UCN were found within the lowest ABR quartiles (10.9% for subcities in the highest quartile and 7.4% unmet needs for subcities in the lowest quartile). Higher subcity ABR was associated with lower city GDP in a graded fashion. Subcities with higher ABR also tended to be in cities with higher homicide rates and larger population rate growth over the 2010–2015 period. The lowest quartile of subcity ABR had larger city population size than the other three quartiles. MMR1 coverage was slightly higher in subcities with higher ABR. All three socioeconomic subcity measures had higher median scores in subcities within the lowest quartiles of ABR and a lower median score among the highest quartiles.

**Table 1:** Country, city and subcity-level characteristics by subcity adolescent birth rate quartile

	First	Second	Third	Fourth	Overall
ABR range	(1.0–43.0)	(43.0–58.6)	(58.6–75.3)	(75.4–246.8)	(1.0–246.8)
Subcities (N)	351	351	351	350	1403
Cities (N)*	105	165	167	130	363
Country-level characteristics					
Per cent unmet contraceptive needs	7.4 (7.1, 10.9)	7.4 (7.4, 10.6)	10.6 (7.4, 10.9)	10.9 (10.6, 10.9)	8.3 (7.4, 10.9)
City-level characteristics					
GDP (US$100)	201 (148, 266)	177 (110, 249)	124 (101, 210)	116 (108, 216)	159 (108, 224)
Population size average (100 000 people)	19.4 (4.5, 6.4)	9.4 (3.3, 37.7)	7.2 (3.3, 31.3)	10.7 (3.0, 31.3)	9.5 (3.4, 37.1)
Per cent population growth (2010–2015)	5.8 (4.4, 8.1)	5.5 (4.1, 6.9)	6.3 (4.3, 8.7)	6.9 (4.5, 8.9)	6.0 (4.3, 8.1)
Homicide rates (per 100 000 people)	6.1 (3.1, 15.0)	16.5 (8.5, 29.6)	18.6 (9.9, 36.1)	18.6 (9.5, 29.0)	16.2 (7.2, 26.3)
Per cent MMR1 coverage	91.5 (89.0, 95.6)	94.6 (89.0, 100.0)	96.0 (90.9, 100.0)	96.1 (92.4, 100.0)	94.7 (89.8, 99.7)
Subcity-level characteristics					
Living conditions score	2.1 (0.7, 2.7)	1.7 (0.3, 2.2)	0.4 (−1.8, 1.6)	−1.9 (−3.6, –0.2)	0.8 (−1.5, 2.0)
Service provision score	1.2 (−0.5, 1.9)	0.5 (−0.8, 1.5)	0.0 (−1.5, 1.1)	0.05 (−1.7, 1.2)	0.4 (−1.8, 1.5)
Education attainment score	0.8 (−0.5, 2.2)	−0.4 (−1.0, 0.4)	−0.9 (-1.6, –0.1)	−1.4 (−2.0, –0.6)	−0.6 (−1.4, 0.4)

We present median, 25th and 75th percentiles, unless otherwise specified.

*ABR quartiles were estimated using subcity-level data, some cities are present in more than one quartile.

ABR, adolescent birth rates; GDP, gross domestic product; MMR1, measles, mumps and rubella.

Table 2 presents rate ratios (RRs) of ABR associated with country, city and subcity features. City GDP and MMR1 vaccine coverage were associated with ABR in unadjusted models but became null in the adjusted model. In adjusted models, ABR was higher in cities with higher homicide rate (RR 1.09; 95% CI 1.06 to 1.11) and population growth (RR 1.02; 95% CI 1.00 to 1.04); lower ABR were observed in subcities with better living conditions (RR 0.95; 95% CI 0.92 to 0.98) and higher educational attainment (RR 0.78; 95% CI 0.76 to 0.79). In sensitivity analyses, higher proportion of women 26–49 with pap smear in the last 3 years was associated with 10% lower ABR in the unadjusted model (RR 0.90; 95% CI 0.83 to 0.97), however, this association weakened and became non-significant in adjusted models (online supplemental table 1).

**Table 2:** Rate ratios for the associations of country, city and subcity-level characteristics with subcity-level adolescent birth rates

	Exposure contrast (SD)	Model 1	Model 2	Model 3	Model 4
		RR (95% CI)	RR (95% CI)	RR (95% CI)	RR (95% CI)
Country-level characteristics					
Per cent unmet contraceptive needs	3.48	1.10 (0.98 to 1.23)	1.10 (0.98 to 1.23)	1.09 (0.99 to 1.21)	1.02 (0.92 to 1.14)
City-level characteristics					
GDP	9526	**0.95** (**0.93 to 0.98**)		0.97 (0.95 to 1.00)	1.01 (0.99 to 1.03)
Average population size	6 110 912	0.97 (0.88 to 1.01)		0.97 (0.92 to 1.02)	0.96 (0.91 to 1.01)
Population growth (2010–2015)	2.84	1.00 (0.97 to 1.03)		1.01 (0.99 to 1.04)	1.02 (1.00 to 1.04)
Homicide rates (per 100 000)	17.96	**1.12** (**1.08 to 1.15**)		**1.10** (**1.07 to 1.13**)	**1.09** (**1.06 to 1.11**)
MMR1 vaccine coverage	11.10	**0.97** (**0.94 to 0.99**)		0.98 (0.95 to 1.00)	0.99 (0.97 to 1.01)
Subcity-level characteristics					
Living conditions score	2.63	**0.75** (**0.73 to 0.77**)			**0.95** (**0.92 to 0.98**)
Service provision score	1.75	**0.85** (**0.84 to 0.87**)			1.00 (0.98 to 1.02)
Education attainment score	1.62	**0.75** (**0.74 to 0.77**)			**0.78** (**0.76 to 0.79**)
Random effects					
Country-level intercept (Var (95% CI))			**0.06** (**0.02 to 0.15**)	**0.04** (**0.02 to 0.12**)	**0.06** (**0.02 to 0.15**)
City-level intercept (Var (95% CI))			**0.03** (**0.03 to 0.05**)	**0.03** (**0.02 to 0.04**)	**0.03** (**0.02 to 0.03**)

All exposure variables were standardised to a mean of 0 and a SD of 1, the exposure contrast refers to the SD which represents a one unit increase in exposure.

Bolded values indicate statistically significant estimates at a p<0.05.

GDP, gross domestic product; MMR, measles, mumps and rubella; SD, Standard Deviation; Var, Variance.

In all the models, the random intercepts for city and country remained statistically significant, suggesting important within country and within city variability. The variance of the city-level intercept decreased as we included predictors at a city and subcity level. The country-level variance decreased when we included the city-level predictors, yet increased again when we included subcity-level predictors.

## Discussion

We examined variation in ABR within and between cities in nine LA countries, and investigated how urban social environment features were associated with ABR. Overall, 37% the variability occurred between countries and 47% occurred between cities, highlighting the importance of city and subcity determinants of ABR. In the fully adjusted models, higher city homicide rates and population growth were associated with higher ABR, while higher subcity education and better living conditions were associated with lower ABR.

Prior research on the associations between the urban environment and ABR has largely focused on neighbourhood social characteristics. Research in HIC found that adolescents living in poorer or ‘socially disorganised’ neighbourhoods tend to have a higher prevalence of adolescent risk behaviours, including lack of contraceptive use and sexual debut at younger ages.^16 29^ Social and economic aspects such as racial/income inequalities, higher unemployment rates and lower education levels are associated with higher ABR in both HIC and LMIC.^15 30^ While studies linking LA urban environments at a city or neighbourhood level to ABR are scarce, national-level and regional-level evidence suggests that countries with lower economic development and higher economic inequalities have higher ABR.^8^ Our study adds to this work by documenting large heterogeneities both between and within cities in LA and by demonstrating strong associations of city characteristics, such as homicide rates and subcity socioeconomic conditions with ABR in LA cities even after adjustment for multiple social environment characteristics.

A novel finding of this study is the association of homicide rates with ABR, where a 1 SD higher city homicide rates was associated with a 9% higher subcity ABR in the fully adjusted model. Previous research on the association between contextual violence and ABR has primarily focused on armed conflict or intimate partner violence as risk factors for adolescent risk behaviours, but the relationship between contextual violence and ABR unrelated to those situations remains largely unexplored.^31 32^ Repeated exposure to community violence is associated with poor health outcomes and adolescent risk behaviours.^33^ Homicide rates are considered a reliable measure of social violence and have been linked to poverty, inequality, impunity, corruption and the presence of organised crime in HIC and LMIC.^34 35^ Factors such as rapid urbanisation and migration, combined with underlying poverty and unstable housing, can potentially exacerbate both crime rates and ABR.^36^ Our findings suggest an association between city violence (proxied by homicide rates) and ABR, even after adjusting for subcity socioeconomic and other city features. One potential explanation for this independent association is that in LA crime is sometimes concentrated in young people increasing stress and disorganisation among the young population.^37^ In addition, perceived adversity and short life expectancy perception have also been associated with early fertility patterns.^38^ It is important to further explore the links between contextual violence and ABR, as confirmation of a causal relation could suggest that violence reduction interventions may have an effect on adolescent pregnancy.

Consistent with prior work showing that neighbourhood socioeconomic context is associated with ABR in HIC,^16^ we found that subcity educational attainment and living conditions were independently and inversely associated with ABR. The absence of individual-level socioeconomic data on births makes it impossible to determine whether these associations reflect true contextual effects or are the manifestation of the individual-level impact of education and economic circumstances. Based on prior work, it is likely that both contextual and individual-level factors are important in shaping ABR.^17 36^ Higher individual education level and better socioeconomic status are related to having better economic opportunities and higher rates of contraceptive use, delaying pregnancy.^39^ Poorer subcity living conditions may reflect marginalisation, poverty and limited access to jobs and healthcare. Subcity educational attainment may reflect access to education/economic opportunity which have been linked to ABR.^40^ Our results suggest that improving living conditions, guaranteeing educational access and reducing economic inequalities could potentially reduce ABR in LMIC cities.

Higher country GDP has been found to be associated with lower ABR.^8^ Work in HIC has found that ABR are lower in neighbourhoods with higher economic growth.^17^ Our findings are consistent with these results, as cities with a higher GDP had lower ABR; however, this association was reduced once we adjusted for other city variables and became non-statistically significant after further adjustment for socioeconomic conditions. These results suggest that the associations we observed with city GDP are, at least in our data, entirely attributable to contextual education and living conditions. Higher city population growth was weakly associated with higher ABR in the fully adjusted model. City population growth may reflect the migration of young people into the city in search of better work opportunities.^41^ Migration or rapid growth may be linked to inequitable access to resources and opportunities, which could also be related to ABR.^4 8^ Understanding the pathways though which city economic development, economic inequality, city size and other factors interact and impact ABR could suggest local policies to prevent ABR.

We found a positive although not statistically significant association between country-level UCN and ABR, this association weakened as we included variables of lower levels but it remained positive, suggesting UCN could be a potential contributor to high ABR in LA. UCN in married women of reproductive age (15–44 years) is one of the progress indicators for the SDGs.^2^ It has been constantly associated with higher rates of adolescent pregnancy at an individual level, and it is predominantly targeted by adolescent pregnancy prevention programmes worldwide.^5^ Despite the wide use of this indicator to monitor family planning programmes, the use of this indicator in the adolescent population has been criticised, given that it is targeted at women of reproductive age (15–44 years) who are married and already had children, excluding young, unmarried and male adolescents.^42^ Similarly, other reproductive healthcare access indicators such as antenatal care or the per cent of deliveries assisted by health professionals, which would have better informed the association of reproductive healthcare access and ABRs, are not specifically targeted to adolescents. In addition, this information is currently available at a country level with limited country-level variability with our sample.^43^ There is a need to improve information related to adolescent contraceptive access that is more inclusive and age appropriate and develop subnational monitoring systems that allow for comparable data disaggregation to inform local policies.

In sensitivity analyses conducted in a subset of cities with available data, we found that an alternative city-level measure of reproductive healthcare access (the percentage of women 26–49 with a pap smear in the sensitivity analysis) was also associated with ABR. As in the case of UCN and MMR1 vaccine coverage, the association was reduced but remained inverse (higher PAP coverage and lower ABR) in fully adjusted models. Although these measures are very imperfect proxies of access to care among adolescents, taken together our results suggest that access to services may also play a role. The fact that associations were weakened after adjusting for socioeconomic indicators is consistent with evidence that women tend to struggle more to get adequate reproductive healthcare services, including contraception and safe abortion, in more marginalised areas.^44 45^ These inequalities in healthcare access were exacerbated by the COVID-19 pandemic.^46^ ABR along with adolescent health overall, other health outcomes such as infant mortality are affected by social and economic inequalities. Often we observe higher rates in areas where the socioeconomic environment tends to be worse.^25^ Considering social inequalities when targeting healthcare access is paramount to design effective interventions to improve health outcomes among the population, especially vulnerable groups such as children, adolescents and women.

Limitations of our analyses include the imperfect measures of violence and healthcare access. We acknowledge that homicide rates do not capture all the contextual violence occurring in a city, such as burglaries, rape or kidnapping; if these other types of violence are important to ABR, using homicides as a proxy could result in a biased estimate of the impact of contextual violence. We included MMR1 vaccine coverage is an imperfect proxy of healthcare access for the adolescent population, which might explain why no association was found in the fully adjusted models. We did not have subnational information on UCN, which usually varies within a country. Rural and urban estimates tend to differ, and access to contraceptives depends largely on government budget and contraception programmes, which differ by region, state or even municipalities.^47 48^ Sensitivity analyses using another imperfect but more spatially refined measure of reproductive healthcare access yielded similar results. Studies with better measures appropriate to adolescents that are also spatially disaggregated are needed to better understand the role of access to healthcare and contraceptives. Studies with better measures appropriate to adolescents that are also spatially disaggregated are needed to better understand the role of access to healthcare and contraceptives. Ideally, we would have preferred to examine adolescent pregnancy rates rather than birth rates, as birth rates exclude abortions.^49^ However, data on adolescent pregnancy rates is not available across our cities and ABR is a widely used indicator to monitor adolescent sexual and reproductive health. UNICEF estimates that all of the countries included in our study have more than 90% national coverage of birth registration,^50^ still, it is possible that some of the cities included have a lower rate which could bias our results. We studied ABR at the subcity level, which did not allow us to examine heterogeneity between neighbourhoods or smaller areas; yet, having information at a subcity level is important to inform local policies. Lastly, our cross-sectional analyses have limitations in drawing causal inferences.

Although abundant research has demonstrated variations across countries in ABR and associations of country characteristics with ABR, our study is among the first to investigate city and subcity differences in ABR and the factors associated with this variability. This is the first study to include compiled and harmonised data on urban environments, and ABR across more than 300 cities in 9 LA countries. Using multilevel models, we were able to describe heterogeneity in ABR across countries and cities and estimate associations of city and subcity features with ABR highlighting the need for locally driven policies. This is also one of the few studies examining the influence of city social environment on ABR across multiple cities in LMIC. This inclusion of city and subcity-level factors is key to the development of local interventions targeting systemic inequalities through social structural changes instead of relying on decontextualised approaches to changing individual behaviour to reduce ABR.

## Conclusion

Our study provides important evidence that social environment factors in cities such as homicide rates and population growth and subcity living conditions and educational attainment are also associated with ABR. The large heterogeneity found within countries and cities highlights the need to prioritise the development of local policies to reduce inequalities in cities. Our findings support the need to consider holistic interventions on the city and subcity social environments to reduce ABR and reach the SDGs. Considering recent increases in violence and the educational and healthcare crises resulting from the COVID-19 pandemic in LMIC, policies that focus on reducing violence rates and educational and economic inequalities are likely necessary to complement and strengthen the impact of ongoing adolescent pregnancy prevention programmes.

## Data Availability

Data may be obtained from a third party and are not publicly available. The SALURBAL project welcomes queries from anyone interested in learning more about its dataset and potential access to data. To learn more about SALURBAL’s dataset, visit https://drexel.edu/lac/ or contact the project at salurbal@drexel.edu.
