# Supplementary material for: Adolescent birth rates and the urban social environment in 363 Latin American cities
Source: BMJ Glob Health. 2022 Oct 17;7(10):e009737. doi: 10.1136/bmjgh-2022-009737 (PMC9577896; doi:10.1136/bmjgh-2022-009737)
Supplement: Supplementary file 1 [file bmjgh-2022-009737supp001.pdf]

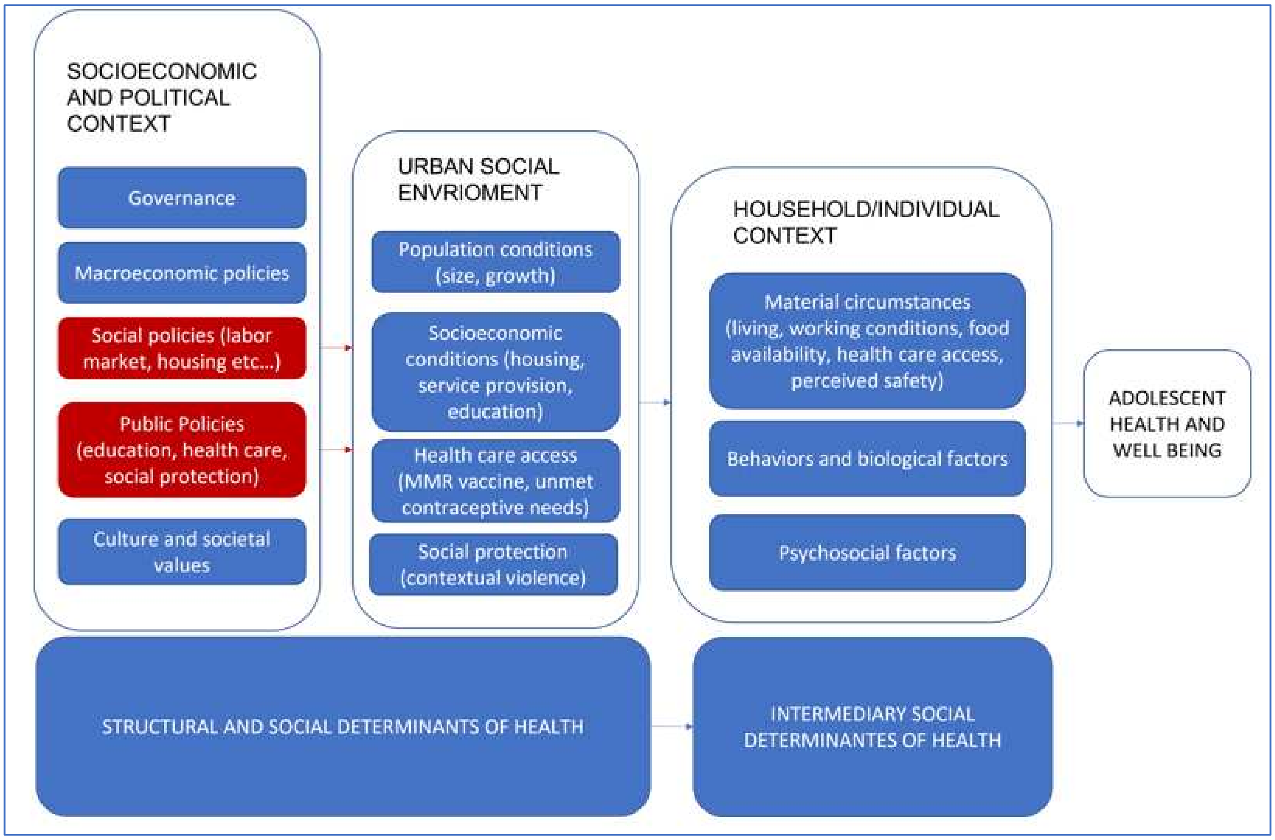

Supplementary figure 1. Conceptual framework for the association of social determinants of health and adolescent health and well being. The boxes highlighted in red correspond to the determinants being explored within this manuscript though the inclusion of disaggregated data of the urban social environment. Adapted from: A Conceptual Framework for Action on the Social Determinants of Health, WHO 2010[18]

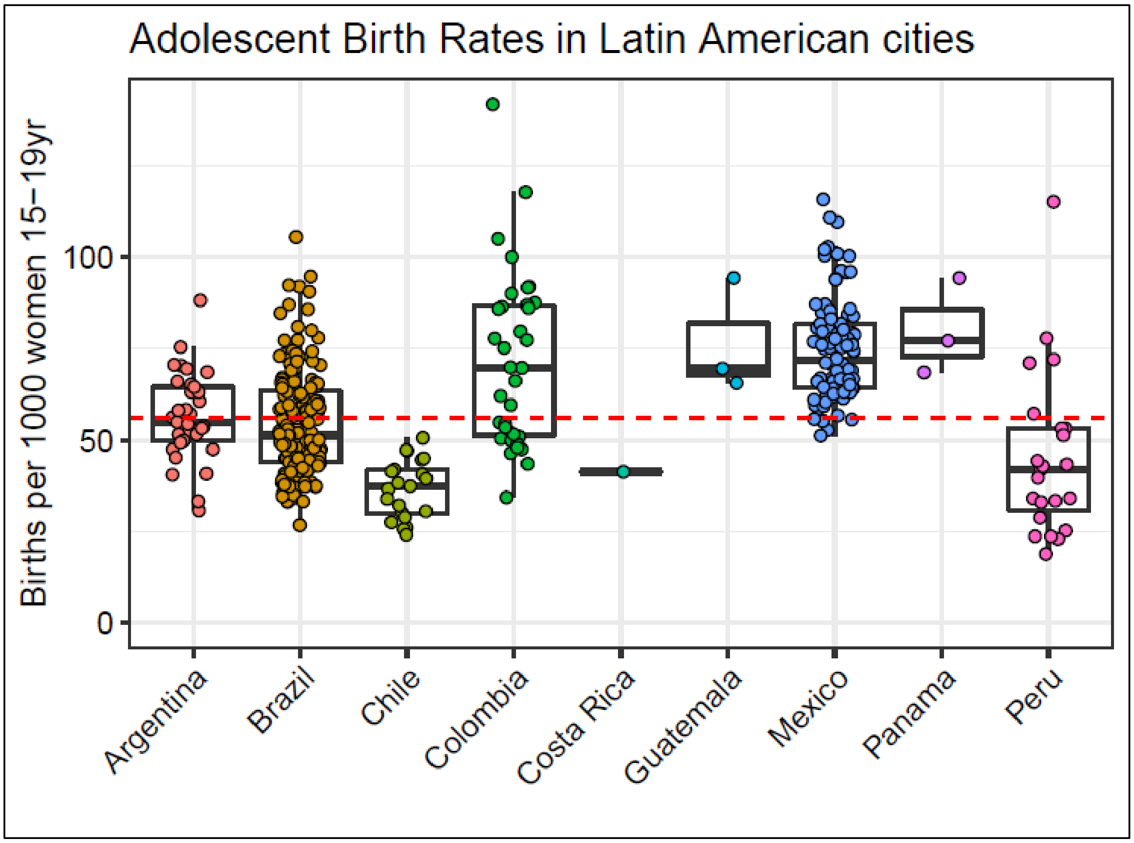

Supplementary figure 2. Adolescent birth rates in Latin American cities distributed by country. Each dot represents a city within a country, 2014-2016 (n=363 cities), and the boxplots show the distribution of adolescent birth rates within countries. Dashed line represents the median adolescent birth rate for all data included in the study (55 births per 1000 women 15-19 years old).

Supplementary table 1. Rate ratios for the associations of country, city, and sub-city level characteristics with sub-city level adolescent birth rates for 176 cities with available data on pap test.

	Exposure Contrast (SD)	Model 1	Model 2	Model 3	Model 4
		RR (95%CI)	RR (95%CI)	RR (95%CI)	RR (95%CI)
**Country level characteristics**					
Percent unmet contraceptive needs	2.53	1.01 (0.90,1.14)	1.01 (0.90,1.14)	0.99 (0.83,1.16)	0.91 (0.79,1.03)
**City level characteristics**					
GDP	10191.22	0.97 (0.94,1.01)		0.99 (0.95,1.03)	1.01 (0.99,1.05)
Average population size	6991453	**0.92 (0.85,1.00)**		0.95 (0.88,1.02)	0.95 (0.89,1.01)
Population growth (2010 to 2015)	2.45	1.00 (0.96,1.04)		1.01 (0.96,1.05)	1.02 (0.98,1.06)
Homicide rates (per 100,000)	18.7	1.04 (0.99,1.10)		1.02 (0.97,1.08)	1.03 (0.99,1.07)
Pap smear in the last 3 years	0.09	**0.90 (0.83,0.97)**		**0.91 (0.84,0.98)**	0.94 (0.88,1.00)
**Sub-city level characteristics**					
Living conditions score	2.73	**0.75 (0.73,0.77)**			0.98 (0.95,1.02)
Service provision score	1.82	**0.85 (0.82,0.87)**			1.00 (0.98,1.03)
Education attainment score	1.71	**0.72 (0.71,0.74)**			**0.74 (0.72,0.76)**
**Random effects**					
Country level intercept (var(95%CI))			**0.06 (0.02,0.20)**	**0.05 (0.01,0.20)**	**0.04 (0.01,0.14)**
City level intercept (var(95%CI))			**0.43 (0.03,0.05)**	**0.03 (0.02,0.05)**	**0.03 (0.02,0.04)**

All exposure variables were standardized to a mean of 0 and a standard deviation of 1, the exposure contrast refers to the standard deviation which represents a one unit increase in exposure.
Bolded values indicate statistically significant estimates at a $p < 0.05$.
GDP- Gross domestic product per capita
